# Non-Canonical Roles of Tau and Their Contribution to Synaptic Dysfunction

**DOI:** 10.3390/ijms221810145

**Published:** 2021-09-20

**Authors:** Giacomo Siano, Chiara Falcicchia, Nicola Origlia, Antonino Cattaneo, Cristina Di Primio

**Affiliations:** 1 Laboratory of Biology, BIO@SNS, Scuola Normale Superiore, Piazza dei Cavalieri 7, 56126 Pisa, Italy; giacomo.siano@sns.it; 2 Institute of Neuroscience, Italian National Research Council, Via Moruzzi 1, 56124 Pisa, Italy; chiara.falcicchia@in.cnr.it (C.F.); nicola.origlia@in.cnr.it (N.O.); 3 European Brain Research Institute (EBRI), Fondazione Rita Levi-Montalcini, Viale Regina Elena 295, 00161 Roma, Italy

**Keywords:** Tau, synaptic dysfunction, Tau oligomers, nuclear Tau, aggregation

## Abstract

Tau plays a central role in a group of neurodegenerative disorders collectively named tauopathies. Despite the wide range of diverse symptoms at the onset and during the progression of the pathology, all tauopathies share two common hallmarks, namely the misfolding and aggregation of Tau protein and progressive synaptic dysfunctions. Tau aggregation correlates with cognitive decline and behavioural impairment. The mechanistic link between Tau misfolding and the synaptic dysfunction is still unknown, but this correlation is well established in the human brain and also in tauopathy mouse models. At the onset of the pathology, Tau undergoes post-translational modifications (PTMs) inducing the detachment from the cytoskeleton and its release in the cytoplasm as a soluble monomer. In this condition, the physiological enrichment in the axon is definitely disrupted, resulting in Tau relocalization in the cell soma and in dendrites. Subsequently, Tau aggregates into toxic oligomers and amyloidogenic forms that disrupt synaptic homeostasis and function, resulting in neuronal degeneration. The involvement of Tau in synaptic transmission alteration in tauopathies has been extensively reviewed. Here, we will focus on non-canonical Tau functions mediating synapse dysfunction.

## 1. Tau Canonical Localization and Functions

Tau protein is a hydrophilic intrinsically disordered protein with a small content of secondary structures [1,2,3]. In physiological conditions, Tau assumes a loop-like conformation in which the N-terminal and C-terminal ends are close when bound to microtubules [4,5]. Two large protein domains, the projection domain and the microtubule binding domain, provide specific properties and functions. The projection domain contains the amino-terminal region (NTD) enriched in acidic residues and the proline-rich region (PRD). The NTD region includes the KKKK sequence involved in heparin binding and the PPXXP/PXXP motifs in the PRD which mediates the interaction of Tau with tubulin and with proteins containing SH3 domains such as the tyrosine kinase Fyn [6,7,8]. The microtubule-binding domain is subdivided into a true tubulin-binding domain with three or four repeats (MTBD) and the acidic carboxy-terminal region (CTD). The repeats are divided in two small regions, a sequence of 18 residues which contains the minimal region with microtubule binding capacity and a second region of 13/14 residues that is a linker region between the repeats [9]. Tau isoforms are able to bind MTs by the MTBD with different affinity depending on the number of repeats: higher for Tau 4R isoforms and lower Tau 3R [10].

Even if Tau in physiological conditions is intrinsically disordered and does not present tertiary structures, pathological events result in the formation of Tau β-structures typical of amyloidogenic proteins in the repeats of MTBD, in particular in R2 and R3, which can assemble by their own in filaments [11]. The pathological process can involve different Tau isoforms and the 3R/4R ratio generate peculiar aggregate structures in different cell types determining distinct tauopathies [12]. Indeed, while in AD both 3R and 4R contribute to aggregation, 4R isoforms are predominant in PSP, CBD, AGD and GGT and 3R are predominant in Pick disease. FTDP-17 is the most variable showing both 3R and 4R or just one isoform [12].

**Figure 1:**
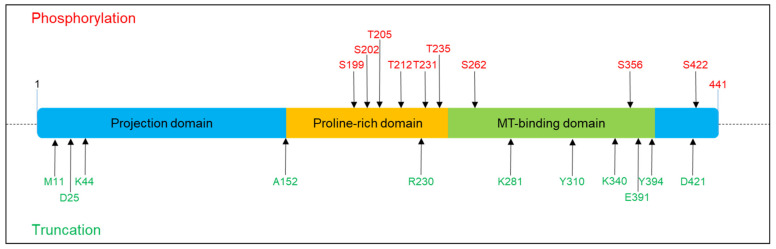
Phosphorylation and truncation sites on Tau protein described in this review. The scheme refers to 2N4R Tau (441 aa). Red colour denotes the phosphorylation sites, and green colour denotes the truncation sites.

Self-aggregation is inhibited by the presence of charged intact N-terminal and C-terminal domains; however, when Tau undergoes post-translational modifications (PTMs), in particular hyperphosphorylation, acetylation and truncation, the conformational structure changes and exposes the sticky repeat regions, which result in the formation of aggregates [13]. Tau PTMs modify the protein altering Tau conformations and properties. Among PTMs, phosphorylation is the most characterized and actually pathology relevant. Seventy-nine putative serine and threonine phosphorylation sites have been identified. Phosphorylation in physiological conditions controls the MT dynamics during normal neurite growth and maturation. In addition, phosphorylation can alter Tau subcellular localization. Phosphorylation of the proline-rich region favours Tau localization mainly in the soma and in the dendritic compartment, whereas, both the dephosphorylation of PRD and the phosphorylation in the C-terminal domain favours Tau localization in the distal axonal region [14,15]. In tauopathies, the uncontrolled phosphorylation events cause an aberrant hyperphosphorylated Tau with increased insolubility and aggregation propensity. All six isoforms hyperphosphorylated at 40 different residues have been identified in aggregates [9,16]. Several critical residues have been identified (Figure 1). Phosphorylation at the AT8 epitope (Ser199/Ser202/Thr205) is sufficient to cause MTs’ remodelling and instability, diminished mitochondrial transport, cell death and neurodegeneration [17]. A similar effect is observed by phosphorylation of Thr212/Thr231/Ser262 [18]. In vitro kinetic studies of the interaction of unphosphorylated and hyperphosphorylated Tau with tubulin identified Ser199/Ser202/Thr205, Thr212, Thr231/Ser235, Ser262/Ser356 and Ser422 as key critical phosphorylation sites that convert Tau to a pathological molecule [19,20,21,22,23]. The phosphorylation of these residues depends on the concerted activity of kinases and phosphatases, for which its equilibrium plays a key role in Tau pathology. Several enzymes involved in phosphorylation pathways have been observed to modulate Tau physiological and pathological behaviour. GSK3b, ERK1/2, Cdk5, JNK, PKA, p38mapK and other kinases are commonly found to interact with Tau protein and NFTs [24,25,26,27]. Different kinases seem to target and phosphorylate specific residues of Tau and have been associated with early or late phosphorylation events. Kinases can induce initial destabilization, as described for the phosphorylation of T231 and S235 mediated by GSK3b, or are involved in aggregation events as described for ERK1/2 [26,28,29,30]. The action of these kinases in normal conditions is countered by the activity of phosphatases, in particular PP2A, PP2B and PP5, which modulate Tau and MTs dynamics. These phosphatases can reduce Tau phosphorylation by direct and indirect mechanisms, and the gain or loss of function in vitro and in vivo induces Tau hyperphosphorylation and aggregation. Accordingly, in AD brains, the reduced level of phosphatases is a peculiar mark and correlates with the aberrant phosphorylation detected during the disease progression [31,32].

In addition to phosphorylation, truncation has been demonstrated to significantly affect Tau stability, resulting in the formation of short Tau species with strong aggregation predisposition [33,34,35,36]. Truncation seems to work synergically with phosphorylation since deprivation of GSK3β prevents the formation of Tau short species [37] (Figure 1). Several enzymes are involved in Tau truncation, and early and late cleavage events have been identified in tauopathies which are associated with Tau species prone to premature nucleation or mature aggregation. Indeed, Tau can be the substrate of caspase3, and the C-terminal truncated Tau becomes prone to forming aggregates [38]. The relevance of Tau fragmentation in neurotoxicity is evident in mice that coexpress truncated and full-length human Tau and showing axonal transport failure, clumping of mitochondria, disruption of the Golgi apparatus and missorting of synaptic proteins. However, halting the expression of truncated Tau determined a functional rescue [39]. Moreover, it has been shown that N-terminally truncated Tau-derived peptides can be extracellularly released and exert neurotoxic actions [40,41].

Another relevant PTM involved in Tau pathology is lysine acetylation, which neutralizes charges in the MTBD thus interfering with Tau interaction with MTs. Hyperacetylation has been detected mostly in intracellular NFTs rather than in pretangles or in extracellular aggregates and anticipates Tau truncation [42,43]. Interestingly, a recent work demonstrated that the overexpression of the hyperacetylated Tau in mice was able to induce a time-dependent progression of neurodegeneration from the entorhinal cortex to the hippocampus that was associated with glial activation and memory deficit [44].

Among PTMs’ glycosylation, glycation, ubiquitination and oxidation are strongly associated with Tau aggregation, in particular with mature NFTs, but their effects on Tau physiological and pathological behaviour are still under investigation.

The downstream effect of these PTMs is Tau aggregation, the hallmark of all tauopathies, although fibrils composition and structure can differ [45,46,47,48,49,50,51,52]. In addition to PTMs, other factors including site-specific mutations or Hsp90 can mediate Tau aggregation [53]. The aggregation process involves several steps, beginning with Tau PTMs, which cause the conformational alteration of the protein, followed by small Tau inclusions with low content in β-sheet structures formed in the process called nucleation; after that, other Tau molecules associate to this core to form neurofibrillary tangles (NFTs). The administration of Tau oligomers in the extracellular medium can induce prion-like homotypic-seeding both in vivo and in vitro, skipping the nucleation process [54,55]. The smallest assembly having these seeding properties is the Tau trimer [56], even if it has been recently demonstrated that monomers isolated from tauopathy brains induce a tauopathy-specific Tau aggregation. This indicates that monomers may possess peculiar pathological characteristics independent from oligomerization and specific for each tauopathy [50,57], possibly related to Tau PTMs. However, the impact that different pathological Tau monomers can have on neuronal homeostasis and pathways is still not completely elucidated and needs further investigation. Moreover, Tau mutated species have proved to be prone to self-aggregation in vivo [53,58,59,60]. It has been extensively reported that Tau aggregation causes cellular stress and damage to neuronal and non-neuronal cells [61]. However, the molecular mechanisms of Tau aggregates toxicity are still debated.

In neuronal cells, Tau is mainly enriched in axons, where it interacts and stabilizes MTs. Each Tau domain mediates tubulin interaction with different mechanisms. The PRD is positively charged and binds the negatively charged MT surface, while the MTBD binds specific pockets in β-tubulin at the inner surface of the MTs. Different repeats of the same MT-binding domain can occupy the β-tubulin pockets of adjacent filaments, thus causing the crosslink of three or four dimers [62,63]. The projection domain outdistances MTs in the axon and may increase the axonal diameter. The acidic Tau N-terminal domain branches away from the MT-surface probably for electrostatic repulsion. By interaction with other cytoskeletal components such as spectrin and actin filaments, the projection domain mediates the interconnection of Tau-stabilized MTs with neurofilaments, thus, restricting the flexibility of MTs lattices [64]. In addition, with tubulin polymerization and stabilization, several pieces of evidence associate Tau with the regulation of axonal transport. Tau does not alter the speed of kinesin along MTs, but it can induce the detachment of cargoes anchored to kinesin [65]. In vitro, Tau knockdown increases the transport velocity in iPSC-derived dopaminergic neurons, suggesting that the presence of the protein may interfere with motor proteins dynamics [66,67]. Moreover, in SHSY5y cells, Tau is also able to bind the p150 subunit of dynactin and is able to stabilize it on MTs, thus, promoting dynein transport [67,68]. Tau is also localized at the axon terminal upon stimulation with NGF and can potentiate NGF and EGF signalling, enhancing the activation of the downstream pathway and modulating neurites extension [69,70].

Tau protein has been detected also in the postsynaptic compartment, where it interacts with PSD-95 in dendritic spines [6]. Upon synaptic activation, Tau delocalizes from the dendritic shaft to spines in cultured neurons and hippocampal slices, suggesting a potential role in the modulation of postsynaptic response [71]. With the PRD region, Tau interacts with the SH3 domains of Fyn, a tyrosine kinase from the Src-family involved in protein trafficking, and this interaction is necessary for Fyn localization at the postsynaptic compartment. Fyn phosphorylates NMDA subunit NR2B, thereby stabilizing its interaction with PSD-95 [7,8].

Moreover, Tau has also been localized close to, or associated to, many organelles, such as ribosomes and mitochondria, or to subcellular compartments such as endoplasmic reticulum and the nucleus. Evidence connecting nuclear Tau with the regulation of gene expression and synaptic functions is significantly growing, as described in the following paragraphs [72,73].

## 2. Impact of Pathogenic Tau Localization

### Tau Mislocalization in the Somato-Dendritic Compartment and Consequent Synaptic Dysfunction

In tauopathies, Tau affinity to tubulin is reduced, resulting in destabilization and disorganization of the axonal cytoskeleton. Moreover, it has been observed that Tau aggregates can sequester not only normal Tau but also the two other major neuronal MAPs, MAP1 and 2 [74]. This loss of function is due to conformational change and misfolding caused by PTMs resulting in aggregation in intracellular fibrillary toxic structures. Remarkably, the number of NFTs correlates with the level of cognitive impairment in tauopathy patients.

The destabilization of axonal MTs affects the plus end-directed transport mediated by kinesin [75]. Altered transport slows down exocytosis and the organelles’ localization; the damage of these mechanisms causes a decrease in lipid and glucose metabolism, ATP synthesis and a loss of Ca^2+^ homeostasis, which result in a distal degeneration process [76,77]. Altogether, these findings raise the hypothesis that the alterations associated with Tau conformational changes may contribute to either pre-synaptic or post-synaptic deficits as early pathological signs of AD. These aspects of the pathological protein behaviour are currently the most studied and characterized by the scientific community. However, recently, preclinical and clinical studies focused on the prevention of cytoskeletal destabilization or on the reduction in Tau aggregates with promising but still unripe results, probably due to the lack of knowledge on unexplored Tau pathological roles [78,79,80,81,82,83]. Indeed, novel non-canonical functions and locations have been discovered, supporting their involvement in neuronal physiology and pathology. Under pathological conditions, Tau is relocalized in the somato-dendritic compartment and in isolated processes of affected neurons [64]. Remarkably, the relocalization of Tau into the somato-dendritic compartment causes the increase in Fyn in dendrites, resulting in toxic hyperexcitability mediated by NMDA receptors. On the contrary, Tau loss prevents deregulated postsynaptic Fyn and, as a consequence, NMDA-dependent excitotoxicity and memory impairment [6,7,8]. The finely tuned interplay between Tau, Fyn and NMDA is relevant for the regulation of the glutamatergic signalling, and it may mediate the process of excitotoxicity in tauopathies. The association between Tau misfolding and synaptic transmission is further supported by the fact that Tau reduction protects from pathological network hyperexcitability in several in vitro and in vivo models, and Tau KO shows impaired LTP [84,85,86]. In addition, the involvement of dendritic Tau in pathology is further underlined by the fact that it is necessary to induce the Aβ-mediated LTP impairment typical of AD conditions, thus supporting that Tau is necessary for and mediates Aβ-induced neurotoxicity in AD [87]. Although abnormal Tau accumulation has an abnormal effect on synapsis, it has also been observed that abnormal signaling at the synapsis can facilitate local tau phosphorylation and translocation [71,88].

To date, thousands of studies have identified Tau protein as a key factor in the pathophysiology of Alzheimer’s disease (AD). Under normal conditions, Tau has been detected in small concentrations at dendrites, but Tau becomes largely missorted into the somato-dendritic compartment and causes synaptic dysfunction in tauopathy conditions [89]. Tau can undergo post-translational changes that result in the characteristic formation of neurofibrillary tangles (NFTs), which are self-assembled paired helical filaments that form inside cell bodies. An early sign of AD neurodegeneration is the presence of NFTs in the entorhinal cortex, which then spread to the hippocampus, amygdala and basal magnocellular complex [90]. Tau modifications that result in NFTs may include hyperphosphorylation, truncation or even acetylation [91]. However, Tau can form not only insoluble deposits but it is also present as non-fibrillar soluble monomeric and oligomeric species that have been found to increase in the brain of AD patients [92,93]. It is worth noting that recent literature pointed at soluble Tau forms (including soluble oligomeric aggregates), but not NFTs or insoluble Tau, as responsible for mediating synaptic dysfunction and toxicity [94]. Therefore, it has been suggested that Tau can be directly involved in the regulation of synaptic function. This was first demonstrated to occur by presynaptic modulation. In particular, presynaptic microinjection of recombinant human Tau protein in the squid giant synapse model was capable of altering synaptic transmission by an increase in transmitter release [95]. This event was mediated by calcium release from intracellular stores and was followed by a reduction in evoked transmitter release. Moreover, this study demonstrated that exogenously injected Tau requires IP3 receptors, GSK3 and Cdk5 activities to block synaptic transmission [95]. This is in agreement with the well-known role of the many kinases that can modulate Tau function contributing to neuronal impairment. On the other hand, the in vivo administration of a calpain inhibitor was capable of decreasing cdk5 activation, thereby diminishing the hyperphosphorylation of Tau, which ameliorated synaptic function and cognition in the 3xTgAD mouse model [96]. Moreover, in the work by Roy et al., the age-related cognitive impairment occurred in the mouse model overexpressing a human mutant Tau form (P301L) known to result in dementia (rTg4510) could be prevented by the administration of a selective p38αMAPK inhibitor, further strengthening the role of this specific kinase in Tau-related pathology [97].

Regarding the abnormal acetylation of Tau protein at two specific lysine residues in transgenic mice, it promotes memory loss and induces an impairment of hippocampal Long-Term Potentiation (LTP) [98].

Studies based on transgenic Tau models contributed to clarifying the relationship between Tau protein and synaptic function either regarding the wild-type or mutated forms. As reported by Dickstein et al., overexpression of the wild-type human Tau in the mouse correlated with a decrease in spines density and altered morphology in cortical neurons [99]. Moreover the expression of human non-mutated Tau results in a decrease in LTP and learning and memory deficits [100].

As reported above and similarly to what has been demonstrated for the role of β-amyloid in AD, soluble small aggregates of Tau protein have gained attention as the pathogenic form inducing synaptic dysfunction. Indeed, soluble Tau was found to be acutely toxic in animal models of tauopathy [101,102,103]. Researchers demonstrated that acute exposure to the extracellular oligomeric form of Tau affects memory and its cellular correlate, LTP [92,104]. It is important to note that these toxic effects of Tau were observed by different preparations of the protein, either Tau derived from transgenic human Tau mice or Tau derived from AD patients [92]. Other studies confirmed an increased amount of oligomeric Tau in the brain of AD patients compared to control, suggesting the possible role of oligomeric Tau as an early biomarker of the disease and the importance of further investigating the biological significance of this particular aggregation state [93].

## 3. Tau Functions in the Nuclear Compartment and Pathological Impact on Synaptic Functions

The presence of Tau in the nuclear compartment was first described in the 1990s in neuroblastoma cells and in human brains [73,105]. It is still unclear how Tau is transported to the nucleus; however, it interacts directly with proteins of the Nuclear Pore Complex (NPC) and has been recently demonstrated to interact with TRIM28, a protein involved in transcriptional regulation and chromatin remodelling, which is able to shuttle Tau to the nucleus [106,107,108]. Concomitant Tau and TRIM28 increased levels have been measured in neuronal nuclei of AD human brains supporting their close dependence in nuclear transport and suggesting a pathological involvement of Tau in chromatin remodelling [108]. Moreover, pathological Tau in primary neurons, in tauopathy models and in AD brains determines a depletion of nuclear Ran and an impairment of the nuclear translocation [109]. Tau contributes in regulating the nuclear homeostasis directly inside the nuclear compartment and indirectly by altering nuclear transport, gene expression and genome structure. In pathological conditions, alterations of the nuclear envelope have been described. Indeed, Tau-dependent aberrant invaginations have been observed in post-mortem patient brains, and this evidence is supported by the fact that pathological Tau induces rearrangement and dysfunction of nuclear lamins, thus causing chromatin modifications, DNA damage and apoptosis as a consequence [106,107].

Tau is involved also in DNA protection, and the interaction between Tau oligomers and p53 in AD mouse models and human brains result in the pathological delocalization of p53 outside the nucleus and to increased susceptibility to DNA damage and neuronal cell death [110]. In a similar manner, a Tau-dependent translocation into the cytoplasm of RNA Polymerase II Subunit RPB1 has been observed in the tauopathy mouse model Tg4510 and in AD human brains, suggesting a pathological role of Tau in transcription alterations [111]. Moreover, pathogenic cytoplasmic Tau induces a reduction in nuclear Ca^2+^ in tauopathy Drosophila model and iPSC-derived hippocampal neurons that causes a CREB depletion from the nucleus and a consequent gene expression alteration which results in neuronal cell death [112]. Recently, Tau involvement in miRNA activity has been reported. Tau interacts with the DEAD box RNA helicase DDX6 involved in translation repression and mRNA decay as well as in the miRNA pathway. The complex Tau/DDX6 increases the silencing activity of the miRNA let-7a, miR-21 and miR-124, affecting their target expression. Indeed, aberrant Tau isoforms are less efficient in modulating the activity of miRNA let-7a, thus impairing miRNA and mRNA homeostasis [113].

The distribution of Tau in the nuclear compartment may depend on the isoform and on its phosphorylation state. In adult mice brains, 2N Tau isoforms are enriched in the chromatin-bound fraction, while 1N Tau isoforms are over-represented in the soluble nuclear fraction. The functional impact of this asymmetric distribution is unclear; it is probable that the presence of 1 or 2 N-terminal sequences alters the affinity for Tau to nuclear cofactors or chromatin even if this point still needs further investigation [114]. Phosphorylation events may impact Tau nuclear distribution. Tau can be found either in a phosphorylated and non-phosphorylated state in the nucleus. Tau localized in the nucleolus is mainly non-phosphorylated, but it can also be phosphorylated in the nucleoplasm in physiological conditions [73,115]. Low levels of phosphorylation increase the affinity of Tau for DNA, while hyperphosphorylation reduces Tau-DNA interaction [116]. The phosphorylation profile of nuclear Tau by mass spectrometry indicates a specific enrichment of Tau phosphorylated at residues T181, S231, S235 and pAT8 in the nuclei of normal cells. Remarkably, the treatment with molecules that cause DNA damage results in an enrichment of non-phosphorylated Tau in the nucleus in order to protect DNA [117]. This evidence suggests a protective role for Tau in the non-phosphorylated form that is supported by observations in mouse models where non-phosphorylated Tau levels are increased in neuronal nuclei after oxidative stress and hyperthermic conditions [115,118]. In addition, since Tau is hyperphosphorylated in pathological conditions, this event might prevent its interaction with chromatin, thus promoting DNA damage that is a typical hallmark of AD and other tauopathies. Moreover, a differential phosphorylation profile is observed for nuclear Tau with aging such as significant phosphorylation of the AT100 epitope, which localizes Tau to heterochromatin, suggesting an epigenetic role for Tau [119]. In the nuclear compartment, Tau is able to directly bind the DNA with the second half of the PRD and the R2 of the MTBD [116,120]. Biophysical studies identified a preference for GC-rich sequences and demonstrated that Tau binding stabilizes the DNA structure when altered by physical stress [121]. Nuclear Tau binds AT-rich α-satellites and colocalizes with nucleolin at the internal periphery of nucleoli [122]. In addition, in primary neurons, Tau also occupies intergenic chromatin regions and promoter regions with a high specificity for GAGA motifs [123]. The mechanisms involved in Tau-mediated chromatin structure, stability or gene expression still need further clarification; however, several functions have been described for nuclear Tau.

Tau emerged as a key player in DNA protection and stability in neuronal cells. It interacts with the minor groove of the double strand DNA helix, stabilizes DNA structure and, as a matter of fact, it increases the dsDNA melting temperature and protects DNA from oxidative stress induced by hydroxyl radicals [120,124]. This protective function was confirmed in mouse models for tauopathies and patients. In tauopathy models, the translocation of Tau in the nuclei is observed under oxidative stress [115,118]. Intriguingly, the presence of pathology-associated Tau mutants results in remarkable chromosome aberrant recombination and aneuploidy in mouse and human samples, suggesting a loss of protective function and supporting the key role of nuclear Tau in genome stability [125,126,127]. Tau also has a key role in chromatin remodelling and gene expression. In Tau knock-out models, several genes show significant gene expression alteration, and some of them regulate themselves [128,129]. Remarkably, in Tau overexpressing drosophila and mice, higher nuclear Tau levels induce a global chromatin relaxation, suggesting that Tau is a factor involved in the loss of heterochromatin observed in AD human brains. This event results in an increase in the transcription of genes physiologically silenced in heterochromatin regions. The impact of this evidence is relevant for the pathology since heterochromatin recovery in the Drosophila restores locomotor damage [130]. Recently, experiments in Drosophila and in human AD and PSP brains demonstrated that altered Tau levels induced dysregulation of transposable elements that cause genomic damage. This effect is due to the loss of heterochromatin described above and the reduction in piwi elements that prevent transposon expression and translocation [119,131,132]. As described above, Tau directly interacts with TRIM28, which mediates its nuclear translocation in physiological and pathological conditions. TRIM28’s crucial role in chromatin remodelling and its relationship with Tau support the hypothesis that TRIM28 may be a relevant factor that mediates Tau-dependent chromatin alterations in tauopathies [108]. Tau might also have a repressive role in gene expression. Indeed, it has been observed that Tau binds promoter regions and represses specific genes [123]. Moreover, Tau interacts with TIP5 in the nucleolus, and Tau depletion results in the increase in 45S-prerRNA synthesis, suggesting that Tau regulates rRNA synthesis in normal conditions [133,134].

The impact of pathological conditions on these nuclear functions are still unclear, but since hyperphosphorylation prevents Tau translocation in the nucleus, this could cause a nuclear Tau depletion and an alteration in ribogenesis, translation and transcription processes as a consequence. In AD brains, a deregulation of SIRT6 and DNA damage results in a pathological increase in Tau acetylation on residue K174, which favours its nuclear translocation. Remarkably, this pathological nuclear Tau species induces an alteration of the expression of genes related to protein synthesis, translation and energy production typically associated with neurodegeneration [135].

## 4. Tau-Mediated Gene Expression and Synaptic Dysfunction

The role of nuclear Tau on gene expression suggests that alterations of Tau physiological state might induce gene deregulation leading downstream to dysfunctions of neuronal processes. Due to the relevant differences in Tau biophysical properties in early and late stages of disease, it is conceivable that alterations in its nuclear functions are distinct during the pathology progression. At the onset of the pathology, Tau nuclear translocation increases, and a significantly increased expression of genes related to the glutamatergic pathway is concomitantly observed, in particular, VGluT1 for the presynaptic terminal and NMDA receptor subunits for the post synapse. Moreover, Tau depletion by RNA interference is followed by the reduction in VGluT1 levels, accounting for a bidirectional relationship between Tau and the *VGluT1* gene expression. Remarkably, experiments of Tau nuclear enrichment employing nuclear localization and export signals demonstrate that the glutamatergic gene expression alteration mainly depends on the nuclear pool of Tau, with no relevant contribution of cytoplasmic Tau [136,137]. Indeed, the Tau-dependent gene alteration results in higher frequency and amplitude of mEPSCs in primary neurons, suggesting a concomitant presynaptic and postsynaptic contribution for the hyperexcitability observed by electrophysiology experiments [136,137]. Neuronal hyperexcitability, due to altered glutamate release, is a typical event at early stages of tauopathies. A higher release of glutamate neurotransmitter is observed in mouse and patients’ brains in concomitance with increased neuronal activity, and this event results in hyperexcitability associated with a higher probability of seizures, synaptic dysfunction and apoptosis, triggering the pathological cascade [138,139,140,141,142]. The close connection between Tau and hyperexcitability is further reinforced by observations in epilepsy models, where the reduction in Tau results in a reduction in seizures [85,142].

As stated above, synaptic dysfunction is partially mediated by cytoplasmic pathological Tau, but these data add a significant contribution to nuclear Tau. Remarkably, a RNAseq analysis comparing differentiated SHSY-5y cells with or without Tau overexpression reveals Tau-dependent global gene alteration (4000 differentially expressed genes), and the GO analyses identifies the glutamatergic pathway as significantly modulated by Tau. In addition, the transcriptome profile of the cellular model has been compared with microarray data of human AD hippocampus at different AD stages. This analysis indicates that the Tau-dependent gene alteration specifically resembles the LMCI profile with no correlation with the EMCI and AD stages (unpublished observations).

In the terminal stages of tauopathies, a reduction in glutamate release and lower excitability can be observed. In these stages, Tau forms amyloidogenic aggregates that represent the typical lesion of Tau pathology [143,144,145]. Experiments on a cellular model mimicking late AD condition showed that aggregation causes a significant reduction in VGluT1 levels, suggesting that nuclear inclusions results in a Tau loss of function [146]. Remarkably, this evidence is further supported by studies in the tauopathy mouse model Tau22, where Tau oligomers in the nuclear compartment have a repressive role on gene expression [123].

Altogether, this evidence supports the hypothesis that synaptic alteration might be caused by the synergistic effect of pathological Tau localized at the synapse and the increase in nuclear Tau that modulates the expression of glutamatergic genes. On the contrary, in late tauopathy conditions, when Tau is mostly aggregated, the glutamate release is lower and synaptic hypoexcitability is observed. This event is related to the formation of repressive Tau amyloidogenic inclusions impairing Tau nuclear function, thus resulting in a reduction in glutamatergic gene levels [123,146]. These stage-specific and pathological Tau-dependent glutamatergic pathway changes correlate genetically and functionally with the synaptic alterations observed in tauopathy human brains. Indeed, the prefrontal cortex (PFC) of AD patients shows higher levels of VGluT1 expression at Braak stages 3 and 4 when Tau is displaced from MTs. On the contrary, low levels of VGluT1 can be observed at Braak stage 6 when Tau is aggregated. A GO analysis reveals that not only VGluT1 is present, but a more general synaptic gene alteration is present specifically at Braak stage 3 and 4, and a significant reduction in gene modulation follows at Braak stage 6 in the PFC of AD patients [146].

Glutamatergic gene alterations in intermediate AD phases might explain the functional synaptic damage that results in hyperexcitability and glutamate release deregulation commonly described in AD. Indeed, higher glutamate release and higher susceptibility to seizures are observed in MCI stages in patients. Late AD stages show synaptic transmission impairment and overall synapse reduction at glutamatergic pathways that could be explained by the gene expression changes in the opposite direction to previous phases [138,139,140,145,147,148,149,150,151].

Altogether, these observations strongly suggest that different tauopathy stages result in peculiar alterations of nuclear Tau properties and functions. Indeed, the different Tau pathological species, destabilized and soluble or aggregated, affect the nuclear Tau function and the synaptic pathway as a consequence. These observations correlate with the stage-specific synaptic alterations that can be observed in tauopathy mouse models and in AD brains. The mechanisms that mediate nuclear Tau gene expression alterations are still unclear, but the repressive role of nuclear Tau oligomers on gene expression has been proposed in tauopathy conditions [123]. Moreover, pathological Tau species alter the chromatin structure, reducing heterochromatin formation in AD brain nuclei. This event causes genomic instability, but it could also induce global gene alteration affecting synaptic functions [132]. Tau is able to bind chromatin by direct (or indirect) interactions, and it is plausible that differences in Tau conformation or PTMs could alter its DNA affinity and its structure. Remarkably, nuclear Tau can interact with key proteins involved in chromatin remodelling, such as TRIM28, and their interaction seems to change during AD progression, suggesting a possible impact on heterochromatin formation and gene expression [108].

The elucidation of these aspects is a central future objective that could result in the discovery of new therapeutic targets preventing tauopathy progression and synaptic damage. However, despite the strong correlation between Tau and AD pathology [58,152], the mechanisms by which this protein is involved in synaptic dysfunction and induces memory impairment still remain elusive [104]. Due to the fact that soluble toxic aggregates can self-propagate and spread throughout the brain, successful therapeutic intervention for AD would benefit from detecting these species before aggregated tangle production and before cognitive impairment becomes evident, with the aim of interfering with the destructive biochemical pathways that this protein initiates. In this view, it is crucial to investigate non-canonical actions of Tau that could contribute to the development and progression of synaptic dysfunction.

## Data Availability

Not applicable.
